# Civil Service Examinations

**Published:** 1892-08

**Authors:** John B. Riley

**Affiliations:** Chief Examiner; Albany, N. Y.


Civil Service Examinations.—An open competitive examination of candidates for Junior Assistant and Female Physicians, to fill vacancies in the medical staff of the several State Hospitals, will be held at rooms of the Civil Service Commission, in Albany, August 24, 1892. Salary of Junior Assistants, $1,400 and board; Female Physicians, $1,200 and board. For further information, apply by mail to Clarence B. Angle, Secretary, Albany, N. Y.

JOHN B. RILEY,
Albany, N. Y., July 20, 1892. *Chief Examiner.*

